# Social, Educational and Medical Aspects after Cataract Surgery of Bilaterally Blind Children in Kinshasa—Perception of Parents and Children

**DOI:** 10.3390/children9111683

**Published:** 2022-11-02

**Authors:** Stefanie Frech, Adrian Hopkins, Astrid Moanda, Janvier Kilangalanga, Rudolf F. Guthoff

**Affiliations:** 1 Department of Ophthalmology, Rostock University Medical Center, 18057 Rostock, Germany; 2 Programme National de Santé Oculaire et Vision (PNSOV), Kinshasa P.O. Box 322, Democratic Republic of the Congo; 3 Réhabilitation à Assise Communautaire (RAC/CBR), Kinshasa P.O. Box 322, Democratic Republic of the Congo; 4 Eye Department, St. Joseph Hospital/CFOAC, Kinshasa P.O. Box 322, Democratic Republic of the Congo

**Keywords:** childhood blindness, bilateral cataract surgery, follow-up, structured interviews

## Abstract

The study investigated the influence of bilateral cataract surgery on the social and educational development of previously bilaterally blind children in Sub-Saharan Africa (SSA), where the prevalence of blindness due to cataract is higher than in high-income countries. The views of both, parents and children, were evaluated with structured interviews based on a newly developed questionnaire related to surgery, follow-up, and family life after surgery. The mean age of the children at interview was 14.4 ± 8.1 years, with 27 females and 26 males. Satisfaction with the outcome of the surgery was reported by 91% of parents. Parents would recommend surgery, because of the children being happy and able to act more independently, with personal, educational and familial factors essentially contributing to the reported satisfaction. The results also showed that 85.0% of children did not wear eyeglasses. Reasons given were mainly cost-related, but also included limited communication between families and health institutions. Providing and maintaining a high-quality and accessible pediatric cataract surgery and healthcare service for follow-up is a major requisite to reduce childhood blindness in SSA. Our study proved the necessity and effectiveness of a community-based rehabilitation program that cares about each individual child, whatever his or her social background.

## 1. Introduction

Globally, cataract is one of the leading causes of childhood blindness besides retinopathy of prematurity and glaucoma, but also one of the most avoidable causes [1,2]. Approximately 1.4 million children in the world are blind, and most of them are living in low- and middle-income countries [3]. Population-based studies in Africa estimated the prevalence of blindness in children between 2 and 8/10,000 children, and cataract accounted for 15–35% of blindness [4]. Compared with high-income countries, the prevalence of blindness due to cataract is 10 times higher in low-income countries [5]. Additionally, the incidence of childhood cataract in Africa is higher than the incidence in industrialized countries. In a total population of 1 million, around 20 children are born with congenital cataract [4]. Including the blind children with developmental cataract (development of cataract within the first year of life due to trauma or disease), the number of blind children due to cataract is even higher. The terms congenital and developmental are mainly used to define the onset of cataract related to vision loss [4]. The causes of childhood blindness vary regionally, and they are mainly based on socio-economic factors and barriers that limit the feasibility of programs to prevent childhood blindness [2]. African studies indicated that blindness caused by un-operated cataract was in the range of 9.1–27.6% of affected children [5]. As barriers to surgery, financial, social, economic and attitudinal reasons are discussed [6].

Vision loss due to cataract within the first year of life increases the likelihood that severe deprivation amblyopia will develop. If the child sees well in the first year of life, then it is likely that the visual pathways develop sufficiently before the onset of cataract [4] to enable successful amblyopia treatment after surgery. Therefore, early diagnosis and treatment are important to prevent irreversible damage and to achieve useful vision after surgery [7].

Our study was located in the Democratic Republic of the Congo (DRC), a low-income country with approximately 90 million inhabitants. Reasonably adequate health care for the population is provided by many facilities, with some external support from governmental or non-governmental organizations. However, there is poor access and high unmet need for health care in large parts of the country, with the result that DRC’s stated health coverage is among the lowest in the world. [8]. Kinshasa, a rapidly growing megacity, is the capital of the Democratic Republic of Congo (DRC) with an estimated population of around 15 million [9]. Within the last two decades, eye care programs and partnerships established a pediatric cataract surgical service in St. Joseph hospital in Kinshasa, including follow-up and community care [10].

The challenge is to follow the treated children over a long period of time, as regular examinations and eyeglass adoption are required to ensure good visual performance. According to the “Blindness and Vision Impairment Collaborators”, cataract surgery and the provision of eyeglasses are among the most cost-effective health-care interventions currently available [11]. The impact of childhood blindness is experienced throughout life and affects social development, education and the chances for adequate employment. These children can pose an emotional and socio-economic challenge to their families, as there is a high likelihood that they will not complete their education and be able to earn a living for themselves [12].

For this reason, this study was performed with the focus on treated children and their guardians who have suffered from cataract and who have experienced life postoperatively. Collecting information and gathering insights including socio-economic aspects of treatment will help to improve the understanding and to give feedback to those who deliver care. Listening to “the patients voice”, as Dean and colleagues name it, produces results that are presented by descriptions and facts from those directly involved [13].

## 2. Materials and Methods

### 2.1. Study Site and Study Population

Children with congenital or development cataract and their caregivers were invited to Saint Joseph’s Hospital in Kinshasa to participate in semi-structured interviews in August 2019. Saint Joseph Hospital is a charity district hospital, located in the Limete district of Kinshasa and owned by the Catholic Archdiocese. Most patients are from low-income families, living in the eastern part of the city, where the majority of households had a lower than average socio-economic level [10]. Families with blind children, identified by the community-based rehabilitation (CBR) program, were referred to the Eye Department of Saint Joseph Hospital, where eye care facilities exist, including surgical facilities, for children with cataract [14]. A total of 67 families participated in the study. Prior to cataract surgery, 14 parents were interviewed (Pre-surgery group), whereas 53 families were interviewed after uni- or bilateral cataract surgery (Post-surgery group).

### 2.2. Study Instrument

We developed semi-structured interview survey instruments with specific questions for parents and children before and after surgery. The main topics included sociodemographic characteristics, information about the parents (literacy status, profession), practical considerations about the hospital visit (travel time to eye clinic, cost of transportation), detection of cataract, the treatment process including surgery and wearing of eyeglasses, beliefs about the surgery (expectations and recommendations), family life, and children’s education.

### 2.3. Data Collection and Analysis

The descriptive, cross-sectional study comprised semi-structured face-to-face interviews, which were conducted in French by a native French speaking ophthalmologist in training. If necessary, the questions were also translated into the local language, Lingala, by a health worker, resident in Kinshasa, in order to avoid communication difficulties and misunderstandings. During the interview, answers and narratives were written down as verbatim as possible. Quantitative data were presented using percentages and mean values ± standard deviation (Excel 2016). Responses to semi-structured open questions were analyzed qualitatively and were subjected to formal thematic analysis. Major themes were identified, with the answers grouped in categories and illustrative quotes noted.

## 3. Results

### 3.1. Characteristics of the Study Population

**Figure 1:**
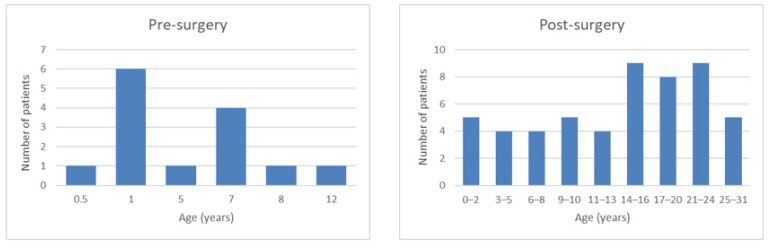
Age distribution of the study sample including 14 patients in the Pre-surgery group (**left**) and 53 patients in the Post-surgery group (**right**).

All participating families lived in the environment of the megacity Kinshasa. In the Pre-surgery group of 14 patients, 7 were male (50%) and the mean age was 4.5 ± 3.7 years, ranging from 8 months to 12 years. In the Post-surgery group comprising 53 patients, 27 were male (51%), and the mean age was 14.4 ± 8.0 years, ranging from 1 to 29 years. Mean age at surgery was 7.9 ± 5.7 years (for 49 children, information on surgery missing for 4) and mean years since surgery were 6.8 ± 6.4 years (for 50 children, information missing for 3). Figure 1 shows the age distribution of both groups of the study sample.

### 3.2. Pre-Surgery Group—Perception of Parents

#### 3.2.1. Visit to the Hospital

Within the Pre-surgery group, in 57%, at least one parent had a profession, and 79% of mothers and 100% of fathers were literate. All families came to the hospital by public transport with a travel time between 0.5 and 3 h. For all, the costs of the trip were reimbursed and managed by the community-based rehabilitation program (CBR). Patients were brought to the hospital for consultation either by their father (15%), mother (50%), both parents (21%), sister (7%) or grandmother (7%). Before coming in on the day of the interview, 50% of patients had seen a doctor (pediatrician, ophthalmologist). Visual disability was detected at different ages, by parents (93%) or health personnel (7%) and by the appearance of white dots. These were observed in the children at an early age, which by the time of the interview had already been some time before, in some cases for several years.

#### 3.2.2. Cataract Surgery

As reported, the treatment process was explained to 64% of the families, although none of them had yet had an appointment for surgery. In total, 93% stated that the CBR program would pay for the surgery, although 64% of parents did not know the costs of the treatment process and the surgery. Slightly more than half of the parents (57%) knew that their child had to wear eyeglasses after surgery but did not know how to pay for the eyeglasses, in contrast to 29% of parents who stated that CBR would pay. Almost all of the parents (93%) knew that their child had to be followed up regularly after surgery, and 79% of parents did not see a problem in coming to the hospital for regular follow-up visits. Asking about parent’s expectations after surgery, 100% believed that their child, in due time, would go to school, help at home and get a job.

### 3.3. Post-Surgery Group—Perception of Parents

#### 3.3.1. Visit to the Hospital

Within the Post-surgery group, in 26%, at least one parent had a profession and 74% of mothers and 83% of fathers were literate. Almost all families (96%) came to the hospital by taxi-bus (one by foot, one unknown) with the same range of travel time between 0.5 and 3 h, as in the Pre-surgery group. The trip was managed by CBR, according to the statement of 98% of families (one unknown). Patients were accompanied by their father (16%), mother (59%), both parents (2%), sister (2%), aunt (8%) or grandmother (13%). Before surgery, visual disability of the patients had been detected by parents (68%), health personnel (2%), CBR members (2%) and others (26%, one unknown). Others were defined as family members (aunt, grandparents), teachers, neighbors or the patient, having realized the visual disability by himself or herself.

#### 3.3.2. Recommendation of Surgery to Others

**Figure 2:**
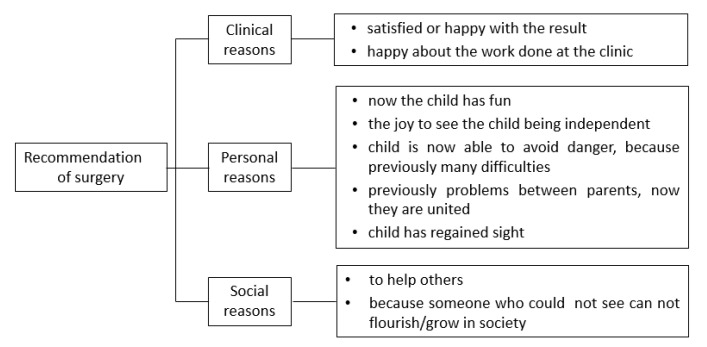
Recommendations of cataract surgery to others.

Most of the parents were satisfied with the results of the surgery (91%), and 83% would recommend the surgery to other families (nine unknown). Reasons for recommendations were grouped into three categories (Figure 2).

#### 3.3.3. Eyeglasses

**Figure 3:**
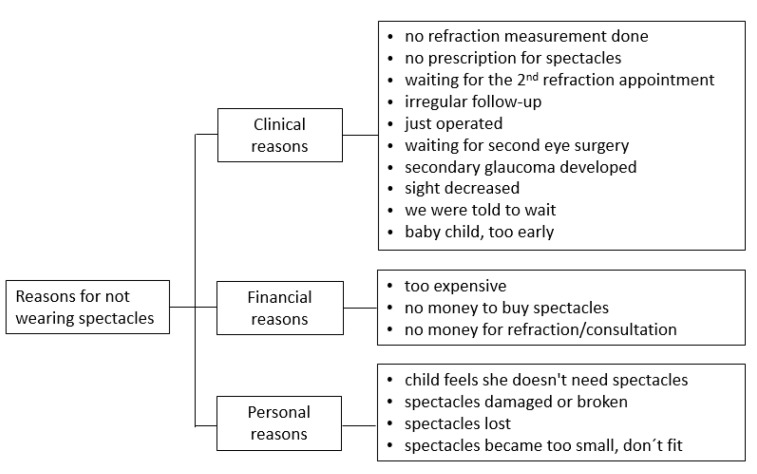
Reasons given by parents why their child did not wear eyeglasses.

According to the parents’ statements, 15% of operated children wore eyeglasses, while 85% did not. Parents’ answers as to why their children did not wear eyeglasses after surgery are summarized in Figure 3.

#### 3.3.4. Follow-Up Examination

**Figure 4:**
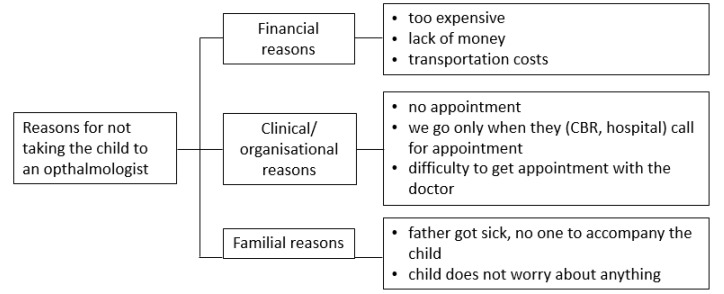
Reasons given by parents for not seeing an ophthalmologist after surgery.

One third of parents (36%) had taken their child to the ophthalmologist, and the last visit was one or more years ago (Figure 4).

#### 3.3.5. Family Life after Surgery

**Figure 5:**
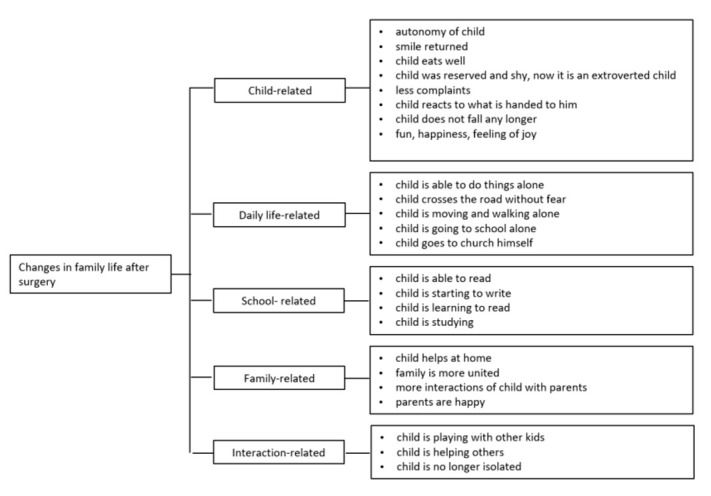
Changes in family life after surgery.

Figure 5 summarizes the answers given by parents concerning the changes in family life after surgery. Being able to see changed life for their children in many aspects, be it child-related or daily life-related (Figure 5).

#### 3.3.6. The Complete Treatment Process at a Glance

Concerning the improvement of the overall treatment process, the answers by parents referred to different aspects including surgery and follow-up. The main issues were the expansion of the program with a free supply of eyeglasses, charge-free postoperative follow-up, follow-up at home, help with anesthesia costs for second eye surgery, postoperative economic help for the families regarding school access and enrollment of their children, and the support to find a job for the parents. The following individual statements were made:

“The first 3 postoperative follow up visits are free, then it is chargeable, which is a problem”

On the other hand, parents were satisfied with the situation and did not expect any changes. They advocated the continuation of the project because many more children needed help and were grateful for the help given:

“keep on helping children”

“do not change anything but go ahead, because many children need help”

### 3.4. Post-Surgery Group—Perception of Children

#### 3.4.1. After the Surgery

After surgery, 98% of children started to feel good, although 25% noted that their eyes hurt due to overexertion or foreign body feeling. Overexertion included reading, writing, playing and watching TV for too long. Almost one third of the children (28%) were afraid of the surgery, and 51% were not (9% were too young to remember, 11% unknown).

#### 3.4.2. School and Education

**Figure 6:**
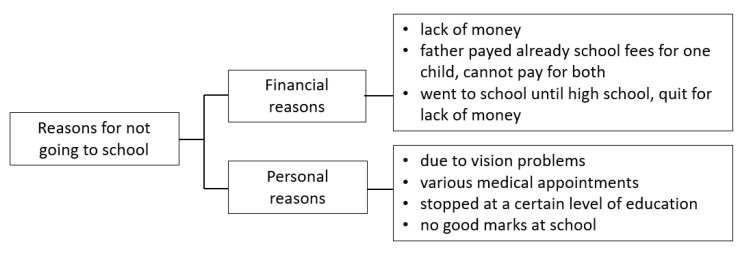
Reasons for not going to school.

After surgery, 77% of the children attended school, including elementary school, secondary school, and university. Parents, organizations and others (priest, cousin) were paying the school fees. Reasons for not going to school stated by the children were multiple and included financial, organizational and personal reasons (Figure 6).

#### 3.4.3. Eyeglasses

**Figure 7:**
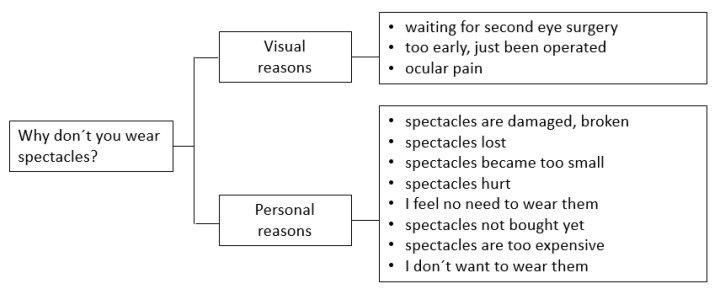
Reasons for not wearing eyeglasses.

Concerning eyeglasses, 16% of children age 6 and older and 13% (n = 53) of all children in this study were wearing them. Reasons for not wearing eyeglasses included personal and health system-related aspects (Figure 7).

#### 3.4.4. Personal Wishes for the Future

Finally, the children were asked about their wishes and dreams for the future. Answers related to learning a profession (doctor, driver, mechanic, nurse, football player, lawyer, accountant, businessman, musician, creating an orphanage, physiotherapist, working in a hospital, becoming a legal advisor, electrician, dressmaker) and wishes for personal life such as helping other people, traveling to Europe, and staying in Kinshasa. Wishes for family and home included having kids, getting married, and building and taking care of the house. Education was also mentioned. Children wanted to finish school, study at the university, or have an apprenticeship.

## 4. Discussion

This study of perceptions of previously bilaterally blind children and their parents provided insights into families’ perspectives and personal and educational aspects about pediatric cataract surgery in SSA. The findings help to better understand families’ attitudes concerning the complex treatment process and may help to improve the way health care is organized and delivered.

### 4.1. Delay between Detection of Cataract and Start of Treatment

Long delays between detection of cataract and the beginning of the treatment process are very common in developing countries [15], which we also experienced in our study. For some families, there was a delay even of several years. As Courtright stated, as soon as a child’s pupil is white, health care personnel must deal with the disease and refer the child to an ophthalmologist, and follow-up must be managed on a regular base throughout childhood [16]. In order to achieve an optimal visual outcome, it is of utmost importance to start amblyopia treatment as soon as possible, which means immediately following surgery together with eyeglass correction based on objective refraction (retinoscopy) and near-vision correction.

It is important to understand parents’ perceptions to find out why some parents sought care for their children while others did not [17]. To gain a better understanding of the delay in surgery in Tanzania, Bronsard and colleagues conducted semi-structured interviews with parents and guardians. Factors that influenced patients’ treatment were local health beliefs about cataract and the ability of health workers to provide information about cataract surgery [18]. In the DRC, too, closer cooperation between families, social workers and the hospital (primary health care staff) is needed to overcome this gap and to facilitate early detection and referral. More comprehensive education would help to increase the number of families being informed about the treatment process and the acceptance of surgical services, as only 64% of parents in our study reported knowing about it. In a Nigerian study, 74.3% of parents were aware of cataract as an eye problem, but had mistaken ideas about the consequences, while in rural Uganda, parental awareness of childhood diseases was high, but the practice of seeking care was low [19]. In Tanzania, an average delay of almost 3 years between recognition of cataract and presentation to hospital for surgery was found to be associated with distance to hospital and a low socio-educational status of the family [20]. In the Kilimanjaro region [18] and in India [21], multifactorial reasons such as ignorance, cost, lack of family support and low socioeconomic status were responsible for delays of 1 to 3 years between recognition of eye problems in children and presentation to hospital.

### 4.2. Barriers Influencing the Treatment Process

All of the individual components of the treatment process, such as local health care structures, health education, surgical procedures, and strictly adherence to follow-up, are essential components and must be taken into account [22]. Nevertheless, there are barriers that influence or even prevent it. In our study, financial aspects played an important role, even if the families were part of a program that covered surgical and indirect costs such as transportation. In order to minimize these negative associations between surgery and associated costs, it is important to inform families in advance. However, even with the provision of affordable or free treatment, other basic barriers may be present, such as fear of surgery, belief in the irreversibility of blindness or lack of information, due to the low education level of parents [10,23]. In addition, attitudes toward children, ignorance of the program, and beliefs about healthcare were discussed in this context [24]. Economic constraints (costs, socioeconomic status) have been identified for The Gambia [25], Malawi [26] and Nigeria [12].

For the institutions involved, knowledge of these barriers is of immense importance when planning programs and health interventions aimed at children’s visual well-being [27]. Muhit presented and discussed barriers in his review and provided suggestions to improve the process, including 10 key messages on childhood cataract that are of utmost relevance for all working in this field [28].

To reach a high proportion of people, mass media campaigns can produce changes in health-related behavior. Having a mobile phone positively influenced the number of referrals for cataract surgery in Rwanda [29]. Factors that contributed to better outcomes, such as availability of needed services and community-based programs, including interventions to support behavior change, were assessed by Wakefield and colleagues [30]. Ntsoane and Oduntan summarized factors affecting the use of ophthalmic services as unavailability, inaccessibility and unaffordability. These included lack of knowledge about a service or the impact of an eye condition. Improving and addressing these factors could influence the prevention of visual impairment worldwide [31].

### 4.3. Parents’ Perceptions Concerning the Recommendation of Surgery to Others and Their Suggestions for Improvement of the Treatment Process

In our study, 91% of parents were satisfied with the results of the surgery, and over 80% would recommend the surgery to others. Parents were pleased to see their child flourishing and gaining sight. In a study investigating factors influencing eye-care seeking behavior of parents, it was found that all parents agreed on the importance of improved vision as a tool to improve further education [27]. This was also a major statement in our study.

Concerning suggestions for improvement, it was stated that a follow-up in the local neighborhood would be of interest. Indeed, one could think about the possibility of arranging follow-up visits in each district. This would simplify the scheduling of follow-up visits in the clinic and relieve the parents from travel costs and travel time. A decentralization of follow-up services was already mentioned by Courtright, in a way that the relevant health care personnel could be trained to provide basic ophthalmic follow-up services [16]. For example, in Nepal, only 8.4% received eye care training, and 14% could measure visual acuity but were keen to receive training [32].

When talking to parents, their concerns, views and beliefs about the disease should be taken seriously and addressed when it comes to the further treatment of their child and a possible upcoming operation [27]. The knowledge and skills of general health workers were investigated in Kenya, Malawi and Tanzania, with the finding that only 8.2% could measure visual acuity and only 56% recognized white pupils as advanced cataract and knew that management included a referral for surgery [33]. A lack of appropriate information regarding childhood blindness and the absence of an appropriate referral protocol contributed to a delay of almost 3 years in Tanzania [34].

Organization of follow-up after surgery remains a challenge and is currently insufficiently integrated into the overall long and complex treatment processes and rehabilitation programs. In the sense of holistic treatment, the existing programs should be re-examined with regard to possible improvements for follow-up. Eriksen and colleagues reported that from five studies performed in Africa and Asia, only 20–40% of children were brought back for the routine follow-up, which is required to refit eyeglasses to assure optimal vision [35]. Compared to children with a short delay in presentation for surgery, children with a long delay in presentation were more likely not to come back for follow-up [35]. One way to improve follow-up was shown by Kishiki and colleagues. They discussed the position of a “childhood blindness and vision coordinator”, who is responsible for a good relationship between parents and the eye care facility and who cares about all aspects during, before and after surgery, including follow-up. In addition, they stated that a systematic approach to improve follow-up is required, rather than single strategies [36]. For Sub-Saharan Africa and South Asia, they found that 59% of facilities reported having such a coordinator, and this was associated with an appropriate follow-up mechanism in place [37].

### 4.4. Wearing Eyeglasses

According to the parents’ statements, 15% of children were wearing eyeglasses. Barriers were financial and ophthalmological ones. Even if the issue is not blindness but refractive errors, wearing eyeglasses is a barrier. In Tanzania, only about 30% [38], and in Nigeria [39], only about 10%, of children with pure refractive errors wore their eyeglasses after prescription. The main barriers in these two studies were costs, access to local optical services, parental concerns about the safety of wearing eyeglasses and “ignorance of refractive status”. On the other hand, two studies conducted in Nigeria showed other factors influencing the decision to buy eyeglasses for children. In one study, frame design, material and quality were the deciding factors [40]; in the second study, parents were aware of the need for eyeglasses for their children but were reluctant to have eyeglasses corrected because they perceived wearing eyeglasses as a stigma [17]. In a review by Burnett and colleagues, interventions and methods were identified to improve eye-care services for school children in low- and middle-income countries. They reported that free eyeglasses led to a higher willingness to wear them, but also cited the need to improve the perception and awareness of treatment options for parents and children and to reduce misunderstandings and stigmatization [41].

We need to emphasize that eyeglasses do not necessarily improve sight immediately, but are the most important tool for successful amblyopia treatment. Costs for eyeglasses should, therefore, be included in the programs, including replacements. One could also consider financial incentives for the successful monitoring of children by health workers.

### 4.5. Strength and Limitations

In health care research, the acceptance of qualitative approaches to gain good results has been growing within the last few years [13]. Listening to patients and their families’ beliefs, which affected their wellbeing, revealed results that could not be measured without this direct interaction. Working closely with the responsible health staff on site in Kinshasa allowed us to ask targeted and relevant questions about treatment to gain a better understanding of the families’ complex situations. Our study excluded families that did not accept our invitation to the interview for different reasons. It could have been possible that these families would have made different statements about the treatment. In addition, it is possible that the information given by the families was biased if they felt that they should have given positive answers and not their own actual thoughts and feelings. Therefore, one has to be careful when interpreting the answers, as they do not necessarily reflect a complete picture of reality. Although the interviews were conducted in French, and a translation into Lingala was arranged, there might have been a language barrier that could have led to misunderstandings or misinterpretation of the questions. As for the children, they may have been distracted and not always have answered the questions correctly.

## 5. Conclusions

In summary, there is evidence that fully financed programs and ongoing projects are of immense importance to further reduce childhood blindness. The number of cataract surgeries is increasing in most countries, and the visual outcome is improving [42]. This fact is a good basis for further development of the pre- and postoperative settings. Nevertheless, governments and international actors must strengthen their efforts to inform communities about disease control programs to improve the quality of health care. Strategies for the management of congenital cataract need to be developed and adapted to regional conditions, with a special emphasis on effective amblyopia treatment.

## Data Availability

The datasets used and/or analyzed during the current study are available from the corresponding author upon reasonable request.
